# Hospitalisation and mortality risk of SARS-COV-2 variant omicron sub-lineage BA.2 compared to BA.1 in England

**DOI:** 10.1038/s41467-022-33740-9

**Published:** 2022-10-13

**Authors:** H. H. Webster, T. Nyberg, M. A. Sinnathamby, N. Abdul Aziz, N. Ferguson, G. Seghezzo, P. B. Blomquist, J. Bridgen, M. Chand, N. Groves, R. Myers, R. Hope, E. Ashano, J. Lopez-Bernal, D. De Angelis, G. Dabrera, A. M. Presanis, S. Thelwall

**Affiliations:** 1 UKHSA COVID-19 National Epidemiology Cell, London, UK; 2 MRC Biostatistics Unit, University of Cambridge, Cambridge, UK; 3 NIHR Health Protection Research Unit for Modelling and Health Economics, MRC Centre for Global Infectious Disease Analysis, Jameel Institute, Imperial College London, London, UK; 4 UKHSA Outbreak Surveillance Team, London, UK; 5 UKHSA Genomics and Public Health Analysis, London, UK; 6 UKHSA HCAI, Fungal, AMR, AMU & Sepsis Division, London, UK; 7 UKHSA COVID-19 Surveillance Cell, London, UK; 8 NIHR Health Protection Research Unit for Respiratory Infections, London, UK; 9 UKHSA Statistics, Modelling and Economics Department, London, UK; 10 UKHSA Joint Modelling Team, London, UK; 11 NIHR Health Protection Research Unit for Behavioural Science and Evaluation, Bristol, UK

**Keywords:** Viral infection, Epidemiology, SARS-CoV-2

## Abstract

The Omicron variant of SARS-CoV-2 became the globally dominant variant in early 2022. A sub-lineage of the Omicron variant (BA.2) was identified in England in January 2022. Here, we investigated hospitalisation and mortality risks of COVID-19 cases with the Omicron sub-lineage BA.2 (*n* = 258,875) compared to BA.1 (*n* = 984,337) in a large cohort study in England. We estimated the risk of hospital attendance, hospital admission or death using multivariable stratified proportional hazards regression models. After adjustment for confounders, BA.2 cases had lower or similar risks of death (HR = 0.80, 95% CI 0.71–0.90), hospital admission (HR = 0.88, 95% CI 0.83–0.94) and any hospital attendance (HR = 0.98, 95% CI 0.95–1.01). These findings that the risk of severe outcomes following infection with BA.2 SARS-CoV-2 was slightly lower or equivalent to the BA.1 sub-lineage can inform public health strategies in countries where BA.2 is spreading.

## Abstract (web summary)

In this cohort study, the authors investigate the risk of severe outcomes following infection from Omicron BA.1 and BA.2 sub-lineages. Using whole genome sequencing and electronic health record data for ~980,000 BA.1 and ~250,000 BA.2 cases in England, they find a slightly lower risk of death and hospitalisation associated with BA.2.

## Introduction

Omicron (B.1.1.529) is currently the globally most prevalent SARS-CoV-2 variant, accounting for >95% of reported variants since late February 2022^1^. The BA.2 sub-lineage of Omicron was first identified in England in January 2022^2^. BA.2 is more transmissible than the previous BA.1 sub-lineage^3^. Small studies have provided inconsistent evidence on the severity of BA.2 relative to BA.1^3–5^.

This study aimed to determine the relative risks of hospitalisation and death for Omicron BA.2 compared to BA.1 in a large national cohort.

## Results

**Fig. 1:**
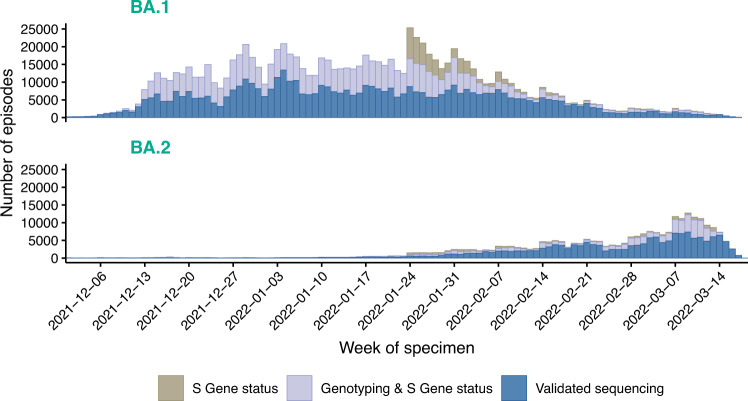
Time series of BA.1 and BA.2 cases in England, 01 December 2021 to 25 March 2022. Descriptive frequencies of the earliest specimen date of COVID-19 cases with Omicron lineages BA.1 (top panel) and BA.2 (bottom panel), in England between 01 December 2021 and 25 March 2022 (*n* = 1,243,212). COVID-19: coronavirus disease 2019. Shading indicates the method of case identification: validated whole genome sequencing, or genotyping in combination with available S-gene status (SGTF or SGTP) for the episode, or from 24th January onwards S-gene status was used in absence of sequencing or genotyping for the episode.

A total of 258,875 BA.2 and 984,337 BA.1 cases with positive specimens between 01 December 2021 and 18 March 2022 (Fig. 1) were included to investigate the risks of the three main outcomes: any hospital attendance within 14 days of a positive test (including admissions), hospital admission within 14 days of positive test where there were ≥2 days of stay and death within 28 days of a positive test. The median age was 38 years (inter-quartile range 25–53) for BA.2 and 35 years (inter-quartile range 22–49) for BA.1 cases; BA.2 cases were more likely to live in less deprived areas or in London, East of England or South East, and had more often received a third vaccine dose, compared to BA.1 cases (Table S1).

**Fig. 2:**
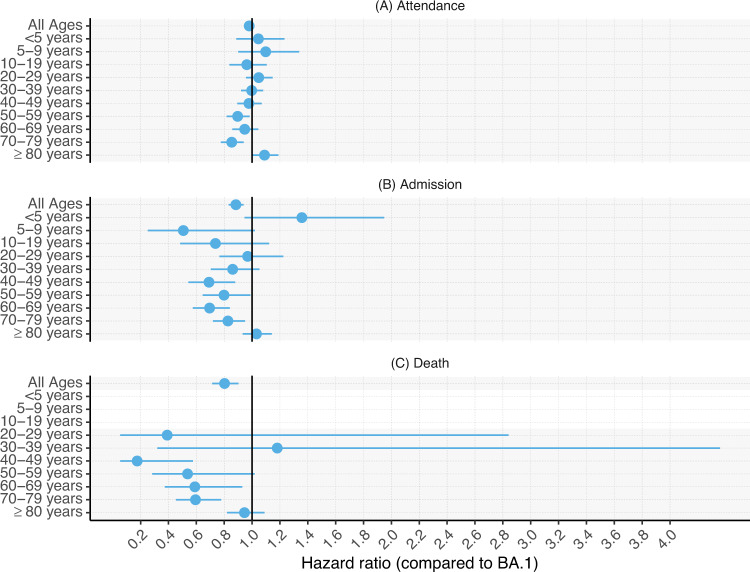
Relative risk of attendance, admission or death, BA.2 versus BA.1 by age group. Risk of hospitalisation and mortality, overall and by age group, for COVID-19 cases with Omicron lineage BA.2 compared to BA.1 in England, 01 December 2021–2025 March 2022. The central measures are adjusted hazard ratios and the errors bars are the corresponding 95% confidence intervals from Cox regression models stratified for exact specimen date, area of residence, age group and vaccination status, and additionally using regression adjustments for within-age-group residual differences in exact age, sex, ethnicity, index of multiple deprivation (IMD) quintile and within-IMD-quintile residual differences in exact IMD rank, and reinfection status. **A** shows risk of any hospital attendance, including admissions, within 14 days of the earliest specimen date in the COVID-19 episode following infection with SARS-CoV-2 lineage BA.2, compared to BA.1. **B** shows the risk of hospital admission within 14 days. **C** shows the risk of death within 28 days. For the death outcome, hazard ratios were not estimated for cases aged <20 years due to small numbers. Likelihood ratio test (LRT) *P* values from two-sided tests for interaction between age group and variant status for attendance, admission, and death are 0.010, 0.0003958, and 0.003 respectively. These explorative tests were not adjusted for multiple comparisons. The adjusted hazard ratio estimates and 95% confidence intervals that the figure is based on are included in Table S3.

Our findings show that the risk of death was lower for BA.2 compared to BA.1 cases (adjusted hazard ratio [HR] = 0.80, 95% CI 0.71–0.90) and the risk of hospital admission was also slightly statistically significantly lower for BA.2 (adjusted HR = 0.88, 95% CI 0.83–0.94). Risk of any hospital attendance was similar between BA.2 and BA.1 (adjusted HR = 0.98, 95% CI 0.95–1.01; Table S2). There were some indications that the risks may differ between age groups, with reduced risks of hospital admission and death for cases with BA.2 compared to BA.1 being specific to adults aged 40–79 years, whilst the risks did not significantly differ between BA.2 and BA.1 for children and for those aged ≥80 years (tests for interaction, all *P* values ≤0.01; Fig. 2; Table S3). However, broad CIs for the HRs in younger age groups complicates drawing conclusions that the risks are age specific.

**Fig. 3:**
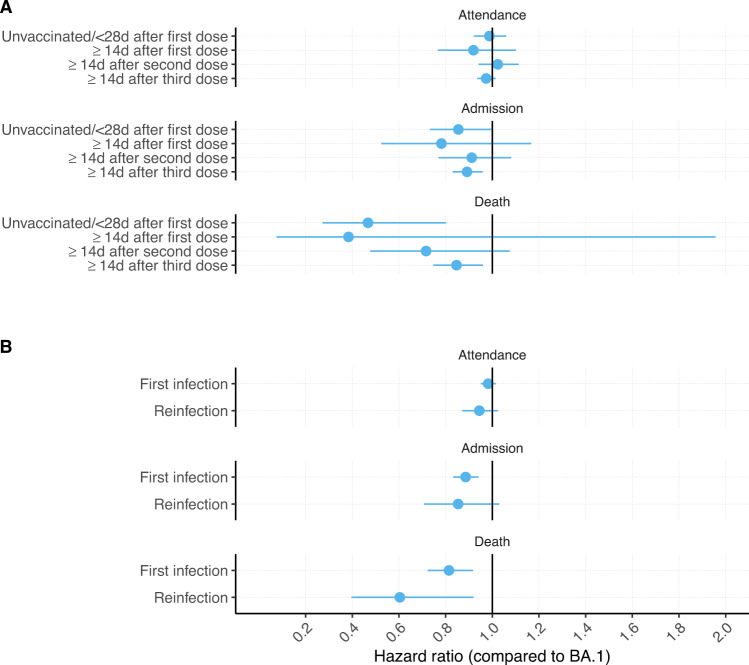
Relative risk of hospital attendance, admission or death, by vaccination status and reinfection status. Risk of hospitalisation and mortality by vaccination status and reinfection status, for COVID-19 cases with Omicron lineage BA.2 compared to BA.1 in England, 01 December 2021–2025 March 2022. The central measures are adjusted hazard ratios and the error bars are the corresponding 95% confidence intervals (CI) from Cox regression models with an interaction term between variant (BA.2 vs BA.1), and vaccination (**A** total *n* = 1,243,212; unvaccinated or ≤28 days after vaccination = 247,748; ≥28 days after first dose = 64,694; ≥14 days after second dose = 294,071; ≥14 days after third dose = 537,822) or reinfection (**B** total *n* = 1,243,212; reinfection = 125,239; first infection = 1,117,973) status. The models were stratified for exact specimen date, area of residence, age group and vaccination status, and additionally using regression adjustments for within-age-group residual differences in exact age, sex, ethnicity, index of multiple deprivation (IMD) quintile and within-IMD-quintile residual differences in exact IMD rank, and reinfection status. LRT *P* values from two-sided tests for interaction between vaccination status and variant status for attendance, admission, and death are 0.65, 0.87, and 0.11 respectively. LRT *P* values from two-sided tests for interaction between reinfection status and variant status for attendance, admission, and death are 0.34, 0.70, and 0.16, respectively. These explorative tests were not adjusted for multiple comparisons. The adjusted hazard ratio estimates and 95% confidence intervals that the figure is based on are included in Table S4.

Adjusted HRs by vaccination and reinfection status showed no significant variation in the risks for BA.2 compared to BA.1, with point estimates indicating lower or similar risks for BA.2 than BA.1 in all subgroups. The point estimate for risk of death for BA.2 compared to BA.1 was somewhat lower for unvaccinated people than those of the other subgroups, but the CIs were wide and overlapped and the corresponding tests for interaction did not indicate significant differences (tests for interaction, all *P* values≥0.11; Fig. 3; Table S4).

Sensitivity analyses examining the effect of differing variant classification methods showed HR estimates were generally higher for the subgroups with non-sequencing-classified variants (Table S5). These were however based on smaller numbers and had much wider CIs than the estimates for the subgroup with sequencing-classified variants, which were consistent with the primary analysis. Alternative definitions of hospitalisation and death endpoints had no substantial effects on the results (Table S6).

We explored the impact of epidemic phase bias (see Methods)^6^. This sensitivity analysis indicated that the risks might be somewhat lower for BA.2 than BA.1 than found in the main analysis (Table S7).

When restricted to different calendar periods based on changes in testing advice (Methods, Table S8), or to a shorter calendar period when the incidence of BA.1/BA.2 was similar (Table S9), the resulting HRs were consistent with and had 95% CIs that overlapped with the main HR estimates.

## Discussion

In this large national study, we found lower or similar risks of severe outcomes for cases with the BA.2 variant compared to BA.1. These risks might vary by age group, with possibly lower risks of mortality and hospital admission for adult BA.2 compared to BA.1 cases aged 40–79, but insignificant differences in the risks for children and for those aged ≥80 years (Fig. 2). We found no significant differences between BA.2 and BA.1 by vaccination or reinfection status, but the precision of the estimates was low in these subgroups.

Results have been mixed in previous reports on BA.2 versus BA.1 severity: a small cohort in France found evidence of a 3.5-fold risk of hospitalisation but based on only 207 BA.2 cases^5^; studies in Denmark^3^; and South Africa^4^ found no significant differences in risk of hospitalisation or death, with relative risks ranging between 0.91 and 1.20, consistent with our findings of at most minor differences in risk between BA.2 and BA.1.

Several SARS-CoV-2 variants have evolved during the pandemic, with inconsistent transmissibility and severity patterns. Omicron BA.1 replaced Delta as the globally dominant variant in early 2022^1^, likely due to greater transmissibility^7^, but is associated with substantially lower severity^8–10^. Both Delta and the previous Alpha (B.1.1.7) variant had higher transmissibility and severity compared to the previous variants^11–13^. It is therefore reassuring that the BA.2 sub-lineage, despite being more transmissible than BA.1^4^, may be associated with no greater risk of severe outcomes than Omicron BA.1. These patterns are analogous with previous findings for the AY.4.2 sub-lineage of the Delta variant, which had higher transmissibility but lower or similar severity risks compared to other Delta sub-lineages^14^.

To prioritise high specificity in the context of lower risks with Omicron compared to previous variants and high vaccine coverage, which might result in higher proportions of incidental admissions, the primary hospital admission definition included inpatient stays where the duration of stay was ≥2 days. This differs from the definitions used in previous variant severity analyses in England, which considered admissions with ≥1 day of inpatient stay^7, 9^; however, sensitivity analyses indicated consistent results between alternative outcome definitions.

This is the largest nationwide study to date to report on BA.2 severity. It includes sufficient numbers to estimate risks by age group, which is important because BA.2 severity has not previously been well described in older age groups^15^. Limitations of the routine surveillance data include reporting lags for hospitalisations and deaths. However, by stratifying on specimen date and region, differential misclassification by variant should not be expected. Cases’ variant status was ascertained based on several different methods; by using a combination of S-gene status, genotyping and sequencing, we could maximise numbers. Sensitivity analyses restricted to cases with variants called by relatively less specific genotyping or S-gene status did not detect reductions in severity between the two lineages, but the primary results were consistent with those for cases with whole-genome sequenced specimens, the most specific method.

There were no data available on some factors that may have affected the risk of hospitalisation or death, such as comorbidities and the use of novel antivirals or monoclonal antibodies. However, there is little reason to expect that these factors are differentially associated with infecting Omicron sub-lineage and they are therefore unlikely to confound the association between sub-lineage and the outcomes. Furthermore, the adjustments for age, sex and deprivation may have partially adjusted for these factors indirectly. The analysis was based on data for test-positive cases whose infecting Omicron sub-lineage was determined. This might lead to selection bias if detection rates or variant calling rates differ by Omicron sub-lineage. However, we are not aware of any data to suggest this.

In conclusion, our study indicates no increased severity of the BA.2 sub-lineage of the SARS-CoV-2 Omicron variant, compared to the previously dominant Omicron BA.1 sub-lineage in England. These findings will be useful in informing public health strategies in countries where BA.2 is spreading.

## Methods

### Case ascertainment

Laboratory-confirmed COVID-19 cases in England with specimen dates between 01 December 2021 and 25 March 2022 were extracted from UKHSA’s Second Generation Surveillance System (SGSS)^16^, and linked to validated whole genome sequencing results available on the Cloud Infrastructure for Big Data Microbial Bioinformatics database^17^ using a unique identifier. PCR genotyping results and S-gene target data were obtained from SGSS.

BA.1 is associated with S-gene target failure (SGTF) and BA.2 with S-gene target positivity (SGTP), but other variants such as Delta (B.1.617.2) are also associated with SGTP. Genotyping could call Omicron variants but not distinguish Omicron sub-lineages. Therefore, the study included: (1) all sequencing-confirmed BA.1/BA.2 cases; (2) before 24th January when Delta variants were still prevalent, cases with SGTF/SGTP status only if genotyping results indicated Omicron; and (3) from 24th January onwards, when the predictive values of SGTF/SGTP to call BA.1/BA.2 were ≥95%^18^, all cases with SGTF/SGTP data that did not already have sequencing/genotyping data (S-gene information available up to 15 March 2022) (Fig. 1).

The cases were further linked to the National Immunisation Management Service for vaccination status; UKHSA’s COVID-19 mortality dataset^19,20^; and NHS Digital’s Emergency Care Data Set and Secondary Uses Service datasets^21^, including data submitted by NHS Trusts up to 8th May 2022, for hospitalisation data.

### Statistical methods

HRs of the outcomes for BA.2 versus BA.1 cases were estimated using Cox regression models. Separate models were fitted for each of the three outcomes. To adjust for confounders, the models were stratified for exact specimen date, area of residence, age group and vaccination status, and additionally used regression adjustments for within-age-group exact age (linear terms), sex, ethnicity, index of multiple deprivation (IMD) quintile and within-IMD-quintile exact IMD rank (linear terms), and reinfection status. We estimated the corresponding HRs by age group, vaccination or reinfection status by fitting secondary models that additionally included interaction terms between variant sub-lineage and each of these potential modifiers. The interactions were tested for significance using likelihood ratio tests.

### Sensitivity analyses

In sensitivity analyses, we examined the effect of differing variant classification methods (Table S5), or alternative definitions of the hospitalisation and death endpoints (Table S6). We explored the impact of epidemic phase bias, which can occur when comparing two virus variants in different phases of growth if infection severity is correlated with time from infection to test. The relative severity between variants of cases that test positive within the same calendar period can then be biased towards overestimated relative risks for a new, more rapidly spreading, variant compared to a previous variant (Table S7)^6^. To assess whether changes in testing guidelines over time affected the association between sub-lineage and severe outcomes we undertook severity analyses, repeating the main analysis limiting cases to three periods: 01 December 2021 to 10 January 2022 (the date at which PCR confirmation of asymptomatic LFT positives stopped), 11 January to 20 February 2022 (when testing in schools ceased) and 21 February to 31 March 2022^22^ (Table S8). A further sensitivity analysis considered a period when the prevalence of the two sub-lineages was similar (14 February 2022 to 27 February 2022^23^, Table S9).

### Software and databases

Data were analysed using R version 4.2.1. SGSS runs SQL Server 2019 (version 15).

### Reporting summary

Further information on research design is available in the Nature Research Reporting Summary linked to this article.

## Supplementary information

Supplementary Information

Reporting Summary

## Data Availability

All data used in this analysis were anonymised, the individual-level nature of the data used risks individuals being identified, or being able to self-identify if the data are released publicly. Requests for access to the underlying source data should be directed to the Office for Data Release (odr@ukhsa.gov.uk) at UKHSA. Details of applying for access to data held by UKHSA can be found at https://www.gov.uk/government/publications/accessing-ukhsa-protected-data/accessing-ukhsa-protected-data and criteria against which applications will be judged can be found at https://www.gov.uk/government/publications/accessing-ukhsa-protected-data/approval-standards. Accessible links for the databases used in this study can be found at: https://sgss.phe.org.uk/Security/Login?ReturnUrl=%2f for SGSS, https://www.climb.ac.uk/ for CLIMB, https://www.scwcsu.nhs.uk/services/nhs-immunisation-management-service/ for NIMS, https://digital.nhs.uk/services/secondary-uses-service-sus for SUS for SUS, https://digital.nhs.uk/data-and-information/data-collections-and-data-sets/data-sets/emergency-care-data-set-ecds for ECDS.

## Code availability

The code used in this analysis is available on GitHub. H.H. Webster, T. Nyberg, M.A. Sinnathamby, N. Abdul Aziz, N. Ferguson, G. Seghezzo, P.B. Blomquist, J. Bridgen, M. Chand, N. Groves, R. Myers, R. Hope, E. Ashano, J. Lopez-Bernal, D. De Angelis, G. Dabrera, A. Presanis, S. Thelwall. Hospitalisation and mortality risk of SARS-CoV-2 variant Omicron sub-lineage BA.2 compared to BA.1, England. Ba2_severity, DOI: 10.5281/zenodo.7043668, 2022.
