# Supplementary material for: Hospitalisation and mortality risk of SARS-COV-2 variant omicron sub-lineage BA.2 compared to BA.1 in England
Source: Nat Commun. 2022 Oct 13;13:6053. doi: 10.1038/s41467-022-33740-9 (PMC9559149; doi:10.1038/s41467-022-33740-9)
Supplement: Supplementary file 1 — Supplementary Information [file 41467_2022_33740_MOESM1_ESM.pdf]

**Supplementary Table S1**: Demographic characteristics, and hospitalisation and death outcomes experienced by cases with SARS-CoV-2 infection with BA.1 or BA.2 lineage, in England between 01 December 2021 and 25 March 2022 (n = 1,243,212).

| | Cases | | | | Hospital attendance within 14 days | | | | Hospital admission within 14 days, ≥ 2 days length of stay | | | | Death within 28 days | | | |
|---|---|---|---|---|---|---|---|---|---|---|---|---|---|---|---|---|
| | BA.2 | | BA.1 | | BA.2 | | BA.1 | | BA.2 | | BA.1 | | BA.2 | | BA.1 | |
| | n | % [a] | n | % [a] | n | % [b] | n | % [b] | n | % [b] | n | % [b] | n | % [b] | n | % [b] |
| Total | 258,875 | 100.00 | 984,337 | 100.00 | 10,843 | 4.19 | 37,626 | 3.82 | 2,962 | 1.14 | 13,323 | 1.35 | 794 | 0.31 | 2,721 | 0.28 |
| Age group | | | | | | | | | | | | | | | | |
| <5 | 5,823 | 2.25 | 20,992 | 2.13 | 528 | 9.07 | 1,649 | 7.86 | 112 | 1.92 | 327 | 1.56 | 0 | 0.00 | 3 | 0.01 |
| 5-9 | 14,720 | 5.69 | 59,485 | 6.04 | 323 | 2.19 | 910 | 1.53 | 23 | 0.16 | 97 | 0.16 | 0 | 0.00 | 1 | 0.00 |
| 10-19 | 26,179 | 10.11 | 127,446 | 12.95 | 561 | 2.14 | 2,156 | 1.69 | 56 | 0.21 | 274 | 0.21 | 0 | 0.00 | 0 | 0.00 |
| 20-29 | 37,792 | 14.60 | 176,391 | 17.92 | 1,276 | 3.38 | 5,394 | 3.06 | 200 | 0.53 | 874 | 0.50 | 2 | 0.01 | 7 | 0.00 |
| 30-39 | 52,493 | 20.28 | 198,676 | 20.18 | 1,684 | 3.21 | 6,266 | 3.15 | 244 | 0.46 | 1,098 | 0.55 | 7 | 0.01 | 18 | 0.01 |
| 40-49 | 43,394 | 16.76 | 155,303 | 15.78 | 1,307 | 3.01 | 4,495 | 2.89 | 157 | 0.36 | 811 | 0.52 | 4 | 0.01 | 42 | 0.03 |
| 50-59 | 36,363 | 14.05 | 119,801 | 12.17 | 1,289 | 3.54 | 4,276 | 3.57 | 221 | 0.61 | 1,121 | 0.94 | 29 | 0.08 | 119 | 0.10 |
| 60-69 | 21,771 | 8.41 | 63,792 | 6.48 | 1,190 | 5.47 | 3,541 | 5.55 | 301 | 1.38 | 1,546 | 2.42 | 52 | 0.24 | 238 | 0.37 |
| 70-79 | 11,751 | 4.54 | 34,152 | 3.47 | 1,216 | 10.35 | 3,876 | 11.35 | 576 | 4.90 | 2,531 | 7.41 | 148 | 1.26 | 548 | 1.60 |
| ≥80 | 8,589 | 3.32 | 28,299 | 2.87 | 1,469 | 17.10 | 5,063 | 17.89 | 1,072 | 12.48 | 4,644 | 16.41 | 552 | 6.43 | 1,745 | 6.17 |
| Sex | | | | | | | | | | | | | | | | |
| Female | 148,736 | 57.45 | 552,679 | 56.15 | 6,407 | 4.31 | 22,132 | 4.00 | 1,545 | 1.04 | 6,876 | 1.24 | 384 | 0.26 | 1,272 | 0.23 |
| Male | 110,139 | 42.55 | 431,658 | 43.85 | 4,436 | 4.03 | 15,494 | 3.59 | 1,417 | 1.29 | 6,447 | 1.49 | 410 | 0.37 | 1,449 | 0.34 |
| Ethnicity | | | | | | | | | | | | | | | | |

| | Cases | | | | Hospital attendance within 14 days | | | | Hospital admission within 14 days, ≥ 2 days length of stay | | | | Death within 28 days | | | |
|---|---|---|---|---|---|---|---|---|---|---|---|---|---|---|---|---|
| | BA.2 | | BA.1 | | BA.2 | | BA.1 | | BA.2 | | BA.1 | | BA.2 | | BA.1 | |
| | n | % [a] | n | % [a] | n | % [b] | n | % [b] | n | % [b] | n | % [b] | n | % [b] | n | % [b] |
| Asian | 24,085 | 9.30 | 88,187 | 8.96 | 863 | 3.58 | 3,328 | 3.77 | 160 | 0.66 | 994 | 1.13 | 18 | 0.07 | 115 | 0.13 |
| Black | 6,394 | 2.47 | 34,362 | 3.49 | 334 | 5.22 | 2,200 | 6.40 | 97 | 1.52 | 799 | 2.33 | 11 | 0.17 | 91 | 0.26 |
| Mixed/Other | 9,900 | 3.82 | 37,846 | 3.84 | 361 | 3.65 | 1,480 | 3.91 | 57 | 0.58 | 360 | 0.95 | 6 | 0.06 | 27 | 0.07 |
| White | 205,619 | 79.43 | 773,275 | 78.56 | 8,417 | 4.09 | 27,570 | 3.57 | 2,374 | 1.15 | 10,122 | 1.31 | 683 | 0.33 | 2,241 | 0.29 |
| Unknown | 12,877 | 4.97 | 50,667 | 5.15 | 868 | 6.74 | 3,048 | 6.02 | 274 | 2.13 | 1,048 | 2.07 | 76 | 0.59 | 247 | 0.49 |
| IMD Quintile | | | | | | | | | | | | | | | | |
| 1 (Most deprived) | 40,407 | 15.61 | 219,564 | 22.31 | 2,001 | 4.95 | 9,985 | 4.55 | 540 | 1.34 | 3,557 | 1.62 | 115 | 0.28 | 645 | 0.29 |
| 2 | 50,731 | 19.60 | 204,316 | 20.76 | 2,246 | 4.43 | 8,575 | 4.20 | 632 | 1.25 | 3,129 | 1.53 | 151 | 0.30 | 589 | 0.29 |
| 3 | 54,914 | 21.21 | 192,049 | 19.51 | 2,310 | 4.21 | 7,210 | 3.75 | 615 | 1.12 | 2,531 | 1.32 | 178 | 0.32 | 541 | 0.28 |
| 4 | 55,581 | 21.47 | 188,437 | 19.14 | 2,243 | 4.04 | 6,404 | 3.40 | 625 | 1.12 | 2,248 | 1.19 | 184 | 0.33 | 509 | 0.27 |
| 5 (Least deprived) | 57,242 | 22.11 | 179,971 | 18.28 | 2,043 | 3.57 | 5,452 | 3.03 | 550 | 0.96 | 1,858 | 1.03 | 166 | 0.29 | 437 | 0.24 |
| Region | | | | | | | | | | | | | | | | |
| East of England | 39,666 | 15.32 | 110,614 | 11.24 | 1,322 | 3.33 | 3,532 | 3.19 | 273 | 0.69 | 1,061 | 0.96 | 87 | 0.22 | 250 | 0.23 |
| Midlands | 37,835 | 14.62 | 183,327 | 18.62 | 1,832 | 4.84 | 6,333 | 3.45 | 531 | 1.40 | 2,305 | 1.26 | 124 | 0.33 | 460 | 0.25 |
| London | 50,310 | 19.43 | 151,789 | 15.42 | 2,154 | 4.28 | 8,004 | 5.27 | 493 | 0.98 | 2,957 | 1.95 | 83 | 0.16 | 473 | 0.31 |
| North East and Yorkshire | 31,105 | 12.02 | 198,264 | 20.14 | 1,174 | 3.77 | 6,195 | 3.12 | 299 | 0.96 | 1,966 | 0.99 | 119 | 0.38 | 521 | 0.26 |
| North West | 33,276 | 12.85 | 165,324 | 16.80 | 1,357 | 4.08 | 6,493 | 3.93 | 388 | 1.17 | 2,420 | 1.46 | 96 | 0.29 | 467 | 0.28 |
| South East | 43,722 | 16.89 | 113,782 | 11.56 | 2,104 | 4.81 | 4,452 | 3.91 | 671 | 1.53 | 1,547 | 1.36 | 205 | 0.47 | 300 | 0.26 |

| | Cases | | | | Hospital attendance within 14 days | | | | Hospital admission within 14 days, ≥ 2 days length of stay | | | | Death within 28 days | | | |
|---|---|---|---|---|---|---|---|---|---|---|---|---|---|---|---|---|
| | BA.2 | | BA.1 | | BA.2 | | BA.1 | | BA.2 | | BA.1 | | BA.2 | | BA.1 | |
| | n | % [a] | n | % [a] | n | % [b] | n | % [b] | n | % [b] | n | % [b] | n | % [b] | n | % [b] |
| South West | 22,961 | 8.87 | 61,237 | 6.22 | 900 | 3.92 | 2,617 | 4.27 | 307 | 1.34 | 1,067 | 1.74 | 80 | 0.35 | 250 | 0.41 |
| Vaccination status | | | | | | | | | | | | | | | | |
| Unvaccinated/<28d after first dose | 59,765 | 23.09 | 247,748 | 25.17 | 2,423 | 4.05 | 9,759 | 3.94 | 502 | 0.84 | 2,785 | 1.12 | 42 | 0.07 | 298 | 0.12 |
| ≥28d after first dose | 10,213 | 3.95 | 54,481 | 5.53 | 385 | 3.77 | 1,770 | 3.25 | 90 | 0.88 | 537 | 0.99 | 12 | 0.12 | 90 | 0.17 |
| ≥14d after second dose | 39,112 | 15.11 | 294,071 | 29.88 | 1,481 | 3.79 | 9,212 | 3.13 | 374 | 0.96 | 3,133 | 1.07 | 80 | 0.20 | 596 | 0.20 |
| ≥14d after third dose | 149,785 | 57.86 | 388,037 | 39.42 | 6,554 | 4.38 | 16,885 | 4.35 | 1,996 | 1.33 | 6,868 | 1.77 | 660 | 0.44 | 1,737 | 0.45 |
| Reinfection status | | | | | | | | | | | | | | | | |
| Reinfection | 27,392 | 10.58 | 97,847 | 9.94 | 890 | 3.25 | 3,070 | 3.14 | 162 | 0.59 | 729 | 0.75 | 41 | 0.15 | 169 | 0.17 |
| First infection | 231,483 | 89.42 | 886,490 | 90.06 | 9,953 | 4.30 | 34,556 | 3.90 | 2,800 | 1.21 | 12,594 | 1.42 | 753 | 0.33 | 2,552 | 0.29 |

SARS-CoV-2: severe acute respiratory syndrome coronavirus 2; IMD; Index of Multiple Deprivation.

[a] Proportion of the overall number of cases with each variant (BA.2 or BA.1) (column-wise percentages)
[b] Proportion of the number of cases with each variant (BA.2 or BA.1) in the demographics sub-groups that experienced the outcome (row-wise percentages)

Cases in study period excluded from study and not shown in table: 67,032 missing NHS number (needed to link vaccination status and hospitalisation data), 14,064 missing data on key covariates, 393 with unusual vaccination dose timing that might reflect immunosuppression (third dose less than 80 days after second dose), and 76 first testing positive post-mortem.

**Supplementary Table S2:** Hazard ratios of risk of severe outcomes in cases with BA2 compared to BA1, crude vs. adjusted model

| Outcome | Crude HR (95% CI) | Adjusted HR (95% CI) [a] |
|---|---|---|
| Hospital attendance (including admission) up to 14 days after positive test | 1.11 (1.09 - 1.14) | 0.98 (0.95 - 1.01) |
| Hospital admission up to 14 days after positive test, ≥ 2 days length of stay | 0.89 (0.86 - 0.93) | 0.88 (0.83 - 0.94) |
| Death within 28 days after positive test | 1.36 (1.26 - 1.47) | 0.80 (0.71 - 0.90) |

HR: Hazard Ratio; CI: Confidence Intervals

[a] Adjusted hazard ratios and 95% confidence intervals (CI) from Cox regression models stratified for exact specimen date, area of residence, age group and vaccination status, and additionally using regression adjustments for within-age-group residual differences in exact age, sex, ethnicity, index of multiple deprivation (IMD) quintile and within-IMD-quintile residual differences in exact IMD rank, and reinfection status.

**Supplementary Table S3**: Hazard ratios of risk of severe outcomes in cases with BA.2 compared to BA.1, overall and by age group, as shown in Figure 2

| Age grouping | Adjusted hazard ratio (95% CI)[a] |
|---|---|
| Any hospital attendance (including admission) up to 14 days after positive test | |
| All Ages | 0.98 (0.95 - 1.01) |
| <5 years | 1.05 (0.89 - 1.23) |
| 5-9 years | 1.10 (0.90 - 1.34) |
| 10-19 years | 0.96 (0.84 - 1.10) |
| 20-29 years | 1.05 (0.96 - 1.15) |
| 30-39 years | 1.00 (0.92 - 1.08) |
| 40-49 years | 0.98 (0.89 - 1.07) |
| 50-59 years | 0.90 (0.82 - 0.98) |
| 60-69 years | 0.95 (0.86 - 1.04) |
| 70-79 years | 0.85 (0.78 - 0.94) |
| ≥ 80 years | 1.09 (1.00 - 1.19) |
| *P-value, test for interaction* | 0.003 |
| Hospital admission up to 14 days after positive test, ≥ 2 days length of stay | |
| **All Ages** | 0.88 (0.83 - 0.94) |
| **<5 years** | 1.36 (0.95 - 1.95) |
| **5-9 years** | 0.51 (0.25 - 1.02) |
| **10-19 years** | 0.74 (0.48 - 1.12) |
| **20-29 years** | 0.97 (0.77 - 1.22) |
| **30-39 years** | 0.86 (0.70 - 1.05) |
| **40-49 years** | 0.69 (0.54 - 0.88) |
| **50-59 years** | 0.80 (0.65 - 0.99) |
| **60-69 years** | 0.69 (0.57 - 0.84) |
| **70-79 years** | 0.83 (0.72 - 0.95) |
| **≥ 80 years** | 1.03 (0.93 - 1.14) |
| ***P-value, test for interaction*** | 0.010 |
| **Death within 28 days after positive test** | |
| All Ages | 0.80 (0.71 - 0.90) |
| 20-29 years | 0.39 (0.05 - 2.84) |
| 30-39 years | 1.18 (0.32 - 4.36) |
| 40-49 years | 0.17 (0.05 - 0.58) |
| 50-59 years | 0.54 (0.28 - 1.02) |
| 60-69 years | 0.59 (0.37 - 0.93) |
| 70-79 years | 0.59 (0.45 - 0.78) |
| ≥ 80 years | 0.95 (0.82 - 1.09) |
| *P-value, test for interaction* | <0.001 |

CI: Confidence interval

[a] Adjusted hazard ratios and 95% confidence intervals from Cox regression models stratified for exact specimen date, area of residence, age group and vaccination status, and additionally using regression adjustments for within-age-group residual differences in exact age, sex, ethnicity, index of multiple deprivation (IMD) quintile and within-IMD-quintile residual differences in exact IMD rank, and reinfection status.

**Supplementary Table S4**: Hazard ratios of risk of severe outcomes in cases with BA.2 compared to BA.1, by vaccination and reinfection status, as shown in Figure 3

| **Sub-group** | **Adjusted hazard ratio (95% CI)[a]** | | |
|---|---|---|---|
| | Any hospital attendance (including admission) up to 14 days after positive test | Hospital admission up to 14 days after positive test, ≥ 2 days length of stay | Death within 28 days after positive test |
| Vaccination status | | | |
| Unvaccinated/<28d after first dose | 0.99 (0.92 - 1.06) | 0.85 (0.73 - 1.00) | 0.47 (0.27 - 0.80) |
| ≥28d after first dose | 0.92 (0.77 - 1.10) | 0.78 (0.52 - 1.17) | 0.38 (0.08 - 1.96) |
| ≥14d after second dose | 1.02 (0.94 - 1.11) | 0.91 (0.77 - 1.08) | 0.72 (0.48 - 1.08) |
| ≥14d after third dose | 0.97 (0.93 - 1.01) | 0.89 (0.83 - 0.96) | 0.85 (0.75 - 0.96) |
| *P-value, test for interaction* | 0.65 | 0.87 | 0.11 |
| Reinfection status | | | |
| First infection | 0.98 (0.95 - 1.01) | 0.89 (0.83 - 0.94) | 0.81 (0.72 - 0.92) |
| Reinfection | 0.94 (0.87 - 1.02) | 0.85 (0.71 - 1.03) | 0.60 (0.40 - 0.92) |
| *P-value, test for interaction* | 0.34 | 0.70 | 0.16 |

CI: Confidence interval

[a] Adjusted hazard ratios and 95% confidence intervals (CI) from Cox regression models with an interaction term between variant (BA.2 vs BA.1), and vaccination or reinfection status. The models were stratified for exact specimen date, area of residence, age group and vaccination status, and additionally using regression adjustments for within-age-group residual differences in exact age, sex, ethnicity, index of multiple deprivation (IMD) quintile and within-IMD-quintile residual differences in exact IMD rank, and reinfection status.

**Supplementary Table S5:** Hazard ratio of hospital admission, attendance, and death after infection with SARS-CoV-2 Omicron sub-lineage BA.2 compared with BA.1, by subgroups based on variant classification method

| **Outcome** | **Adjusted Hazard Ratio (95% CI)[a]** | | | |
|---|---|---|---|---|
| | Primary Analysis | Subgroup | | |
| | | Variant classification by whole-genome sequencing | Variant classification by genotyping and SGTF status | Variant classification by SGTF status alone[b] |
| Any hospital attendance (including admission) up to 14 days after positive test | 0.98 (0.95 - 1.01) | 0.98 (0.95 - 1.02) | 0.98 (0.90 - 1.07) | 0.98 (0.84 - 1.15) |
| Hospital admission up to 14 days after positive test, ≥ 2 days length of stay | 0.88 (0.83 - 0.94) | 0.88 (0.82 - 0.93) | 1.00 (0.76 - 1.32) | 1.54 (0.91 - 2.61) |
| Death within 28 days after positive test | 0.80 (0.71 - 0.90) | 0.79 (0.70 - 0.89) | 1.23 (0.71 - 2.13) | 1.04 (0.32 - 3.43) |

SARS-CoV-2: severe acute respiratory syndrome coronavirus 2; SGTF: S-Gene Target Failure Status

[a] Adjusted hazard ratios and 95% confidence intervals (CI) from Cox regression models stratified for exact specimen date, area of residence, age group and vaccination status, and additionally using regression adjustments for within-age-group residual differences in exact age, sex, ethnicity, index of multiple deprivation (IMD) quintile and within-IMD-quintile residual differences in exact IMD rank, and reinfection status.

[b] SGTF status alone was only included from 24th January onwards, from which point the positive predictive value (PPV) of this test alone was found to be high enough to designate the SARS-CoV-2 infection as BA.2 or BA.1.

**Supplementary Table S6:** Hazard ratio of hospital admission, attendance, and death after infection with SARS-CoV-2 Omicron sub-lineage BA.2 compared with BA.1, with differing outcome measurement criteria

| | **Adjusted HR (95% CI)** |
|---|---|
| Hospital attendance (including admissions) | |
| Any hospital attendance (including admission) up to 14 days after positive test | 0.98 (0.95 - 1.01) [a] |
| Hospital attendance and excluding injury | 0.99 (0.96 - 1.02) |
| Hospital attendance up to 14 days after, or 1 day prior to positive test | 0.98 (0.95 - 1.01) |
| Hospital attendance up to 14 days after, or 1 day prior to positive test and excluding injury | 0.99 (0.96 - 1.02) |
| Hospital attendance excluding day of test | 0.99 (0.95 - 1.03) |
| Hospital attendance excluding day of test and excluding injury | 1.00 (0.96 - 1.04) |
| Hospital attendance up to 14 days after, or 1 day prior to positive test, excluding those released on the same day | 0.98 (0.95 - 1.01) |
| Hospital attendance up to 14 days after, or 1 day prior to positive test, excluding those released on the same day and excluding injuries | 0.99 (0.96 - 1.02) |
| Hospital admission | |
| Hospital admission up to 14 days after positive test, ≥ 2 days length of stay | 0.88 (0.83 - 0.94) [a] |
| Hospital admission up to 14 days after positive test | 0.93 (0.89 - 0.97) |
| Hospital admission and any COVID-19 ICD10 code | 0.90 (0.85 - 0.96) |
| Hospital admission and excluding injury | 0.94 (0.89 - 0.98) |
| Hospital admission and length of stay ≥ 1 day | 0.93 (0.88 - 0.97) |
| Hospital admission via A&E only | 0.93 (0.87 - 0.99) |
| Hospital admission up to 14 days after, or 1 day prior to positive test | 0.93 (0.89 - 0.97) |
| Hospital admission up to 14 days after, or 1 day prior to positive test and excluding injury | 0.94 (0.90 - 0.98) |
| Hospital admission excluding day of test | 0.94 (0.88 - 0.99) |
| Hospital admission excluding day of test and excluding injury | 0.94 (0.88 - 1.00) |
| Hospital admission up to 14 days after, or 1 day prior to positive test, excluding those released on the same day | 0.93 (0.89 - 0.97) |
| Hospital admission up to 14 days after, or 1 day prior to positive test, excluding those released on the same day and excluding injuries | 0.94 (0.90 - 0.98) |
| Death | |
| Death within 28 days after positive test | 0.80 (0.71 - 0.90) [a] |
| Death within 14 days after positive test | 0.82 (0.71 - 0.95) |
| Death within 60 days after positive test | 0.80 (0.73 - 0.89) |
| Death within 28 days after positive test and COVID-19 on death certificate | 0.84 (0.72 - 0.98) |
| Death within 14 days after positive test and COVID-19 on death certificate | 0.85 (0.71 - 1.03) |
| Death within 60 days after positive test and COVID-19 on death certificate | 0.84 (0.73 - 0.97) |

SARS-CoV-2: severe acute respiratory syndrome coronavirus 2; COVID-19; coronavirus disease; ICD10: 10th revision of the International Statistical Classification of Diseases and Related Health Problems (ICD);

[a] Primary analysis

**Supplementary Table S7:** Estimation of the effect of epidemic phase bias on the analysis of risk of hospitalisation and mortality, overall and by age group, for COVID-19 cases with Omicron lineage BA.2 compared to BA.1 in England, 01 December 2021 – 25 March 2022

| | **HR (95% CI)** | | | | |
|---|---|---|---|---|---|
| Age Group | 0 days (primary analysis) | 1 day[a] | 2 days[a] | 3 days[a] | 4 days[a] |
| Any hospital attendance (including admission) up to 14 days after positive test | | | | | |
| All Ages | 0.98 (0.95 - 1.01) | 0.90 (0.87 - 0.93) | 0.81 (0.79 - 0.84) | 0.74 (0.72 - 0.76) | 0.68 (0.66 - 0.70) |
| <5 years | 1.05 (0.89 - 1.23) | 0.97 (0.82 - 1.14) | 0.90 (0.76 - 1.05) | 0.84 (0.72 - 0.99) | 0.74 (0.63 - 0.87) |
| 5-9 years | 1.10 (0.90 - 1.34) | 1.01 (0.83 - 1.23) | 0.94 (0.77 - 1.15) | 0.82 (0.67 - 1.00) | 0.76 (0.62 - 0.93) |
| 10-19 years | 0.96 (0.84 - 1.10) | 0.87 (0.76 - 1.00) | 0.77 (0.67 - 0.89) | 0.68 (0.59 - 0.79) | 0.63 (0.54 - 0.72) |
| 20-29 years | 1.05 (0.96 - 1.15) | 0.97 (0.88 - 1.06) | 0.87 (0.79 - 0.95) | 0.80 (0.73 - 0.88) | 0.71 (0.65 - 0.78) |
| 30-39 years | 1.00 (0.92 - 1.08) | 0.90 (0.83 - 0.97) | 0.81 (0.75 - 0.88) | 0.74 (0.68 - 0.80) | 0.67 (0.62 - 0.73) |
| 40-49 years | 0.98 (0.89 - 1.07) | 0.88 (0.81 - 0.97) | 0.81 (0.74 - 0.89) | 0.74 (0.67 - 0.81) | 0.67 (0.62 - 0.74) |
| 50-59 years | 0.90 (0.82 - 0.98) | 0.83 (0.75 - 0.91) | 0.73 (0.67 - 0.81) | 0.66 (0.60 - 0.72) | 0.61 (0.55 - 0.67) |
| 60-69 years | 0.95 (0.86 - 1.04) | 0.87 (0.78 - 0.95) | 0.79 (0.72 - 0.87) | 0.72 (0.65 - 0.79) | 0.65 (0.59 - 0.72) |
| 70-79 years | 0.85 (0.78 - 0.94) | 0.78 (0.71 - 0.86) | 0.72 (0.66 - 0.80) | 0.66 (0.60 - 0.72) | 0.63 (0.57 - 0.69) |
| ≥ 80 years | 1.09 (1.00 - 1.19) | 1.01 (0.92 - 1.10) | 0.93 (0.85 - 1.01) | 0.84 (0.77 - 0.92) | 0.80 (0.73 - 0.87) |
| Hospital admission up to 14 days after positive test, ≥ 2 days length of stay | | | | | |
| All Ages | 0.88 (0.83 - 0.94) | 0.82 (0.78 - 0.87) | 0.75 (0.71 - 0.80) | 0.69 (0.65 - 0.73) | 0.64 (0.60 - 0.68) |
| <5 years | 1.36 (0.95 - 1.95) | 1.24 (0.87 - 1.78) | 1.20 (0.84 - 1.70) | 1.22 (0.85 - 1.73) | 1.06 (0.74 - 1.52) |
| 5-9 years | 0.51 (0.25 - 1.02) | 0.48 (0.24 - 0.97) | 0.43 (0.21 - 0.88) | 0.41 (0.20 - 0.83) | 0.37 (0.18 - 0.76) |
| 10-19 years | 0.74 (0.48 - 1.12) | 0.65 (0.43 - 0.99) | 0.64 (0.42 - 0.98) | 0.56 (0.37 - 0.86) | 0.52 (0.33 - 0.79) |
| 20-29 years | 0.97 (0.77 - 1.22) | 0.94 (0.74 - 1.19) | 0.84 (0.67 - 1.07) | 0.75 (0.59 - 0.95) | 0.69 (0.54 - 0.87) |
| 30-39 years | 0.86 (0.70 - 1.05) | 0.78 (0.64 - 0.95) | 0.69 (0.56 - 0.85) | 0.64 (0.52 - 0.78) | 0.60 (0.49 - 0.73) |
| 40-49 years | 0.69 (0.54 - 0.88) | 0.62 (0.49 - 0.79) | 0.57 (0.45 - 0.73) | 0.51 (0.40 - 0.65) | 0.46 (0.36 - 0.59) |
| 50-59 years | 0.80 (0.65 - 0.99) | 0.74 (0.60 - 0.92) | 0.63 (0.51 - 0.79) | 0.62 (0.50 - 0.76) | 0.56 (0.45 - 0.70) |
| 60-69 years | 0.69 (0.57 - 0.84) | 0.63 (0.52 - 0.76) | 0.60 (0.49 - 0.72) | 0.55 (0.45 - 0.66) | 0.49 (0.41 - 0.59) |
| 70-79 years | 0.83 (0.72 - 0.95) | 0.78 (0.68 - 0.90) | 0.72 (0.63 - 0.83) | 0.66 (0.57 - 0.75) | 0.62 (0.54 - 0.71) |
| ≥ 80 years | 1.03 (0.93 - 1.14) | 0.97 (0.88 - 1.08) | 0.89 (0.81 - 0.99) | 0.81 (0.73 - 0.89) | 0.76 (0.69 - 0.84) |
| Death within 28 days after positive test | | | | | |
| All Ages | 0.80 (0.71 - 0.90) | 0.78 (0.70 - 0.88) | 0.74 (0.66 - 0.83) | 0.71 (0.63 - 0.79) | 0.66 (0.59 - 0.75) |
| <10 years | - | - | - | - | - |
| 10-19 years | - | - | - | - | - |
| 20-29 years | 0.39 (0.05 - 2.84) | 0.46 (0.06 - 3.44) | 0.58 (0.06 - 5.66) | 0.41 (0.06 - 2.95) | 0.43 (0.06 - 3.03) |
| 30-39 years | 1.18 (0.32 - 4.36) | 1.27 (0.34 - 4.70) | 0.98 (0.27 - 3.50) | 1.06 (0.30 - 3.66) | 1.04 (0.31 - 3.47) |

| | | | | | |
|---|---|---|---|---|---|
| 40-49 years | 0.17 (0.05 - 0.58) | 0.19 (0.06 - 0.64) | 0.18 (0.05 - 0.60) | 0.13 (0.03 - 0.49) | 0.15 (0.04 - 0.58) |
| 50-59 years | 0.54 (0.28 - 1.02) | 0.53 (0.28 - 1.01) | 0.41 (0.21 - 0.77) | 0.50 (0.26 - 0.94) | 0.38 (0.20 - 0.72) |
| 60-69 years | 0.59 (0.37 - 0.93) | 0.56 (0.36 - 0.89) | 0.54 (0.34 - 0.86) | 0.57 (0.36 - 0.91) | 0.50 (0.32 - 0.79) |
| 70-79 years | 0.59 (0.45 - 0.78) | 0.61 (0.47 - 0.80) | 0.57 (0.43 - 0.75) | 0.53 (0.41 - 0.70) | 0.52 (0.39 - 0.68) |
| ≥ 80 years | 0.95 (0.82 - 1.09) | 0.90 (0.78 - 1.04) | 0.86 (0.75 - 0.99) | 0.81 (0.71 - 0.93) | 0.77 (0.67 - 0.88) |

HR: Hazard Ratio; CI: Confidence Intervals

[a] A sensitivity analysis where a proxy date for the date of infection is created and adjusted for, instead of the date of positive test. The proxy date is created by shifting the date of positive test for cases who experienced one of the severity outcomes by an assumed number of days, corresponding to the assumed difference in the number of days from infection to positive test between cases who do not experience severe disease and those who do. Because this difference is unknown, we assessed the impact on the results by assuming scenarios where the difference was between 1 and 4 days. As expected, all scenarios resulted in lower HRs for BA.2 compared to BA.1 compared to the primary analysis.

**Supplementary Table S8:** Hazard ratios of risk of severe outcomes in cases with BA2 compared to BA1, within different subgroups by testing periods in England, 01 December 2021 – 25 March 2022

| Specimen date period | HR (95% CI) [a] | | |
|---|---|---|---|
| | Any hospital attendance (including admission) up to 14 days after positive test | Hospital admission up to 14 days after positive test, > 1 day length of stay | Death within 28 days after positive test |
| 01 December 2021 - 10 January 2022 | 0.82 (0.69 - 0.98) | 0.63 (0.43 - 0.92) | - |
| 11 January 2022 - 20 February 2022 | 1.00 (0.95 - 1.04) | 0.90 (0.82 - 0.98) | 0.88 (0.73 - 1.05) |
| 21 February 2022 - 31 March 2022 | 0.98 (0.93 - 1.03) | 0.89 (0.82 - 0.97) | 0.78 (0.67 - 0.91) |

HR: Hazard Ratio; CI: Confidence Intervals

[a] Adjusted hazard ratios and 95% confidence intervals (CI) from Cox regression models with an interaction term between variant (BA.2 vs BA.1), and testing period.

The models were stratified for exact specimen date, area of residence, age group and vaccination status, and additionally using regression adjustments for within-age-group residual differences in exact age, sex, ethnicity, index of multiple deprivation (IMD) quintile and within-IMD-quintile residual differences in exact IMD rank, and reinfection status.

**Supplementary Table S9:** Hazard ratios of risk of severe outcomes in cases with BA2 compared to BA1, comparing between inclusion of all cases between 01 December 2021 – 25 March 2022 and inclusion of cases between 14 February 2022 – 27 February 2022

| | HR (95% CI) [a] | | |
|---|---|---|---|
| | Any hospital attendance (including admission) up to 14 days after positive test | Hospital admission up to 14 days after positive test, > 1 day length of stay | Death within 28 days after positive test |
| All observations | 0.98 (0.95 - 1.01) | 0.88 (0.83 - 0.94) | 0.80 (0.71 - 0.90) |
| Restricted to 14 February - 27 February 2022 | 0.94 (0.89 - 1.00) | 0.83 (0.75 - 0.93) | 0.69 (0.57 - 0.84) |

HR: Hazard Ratio; CI: Confidence Intervals

[a] Adjusted hazard ratios and 95% confidence intervals (CI) from Cox regression models stratified for exact specimen date, area of residence, age group and vaccination status, and additionally using regression adjustments for within-age-group residual differences in exact age, sex, ethnicity, index of multiple deprivation (IMD) quintile and within-IMD-quintile residual differences in exact IMD rank, and reinfection status.
